# 2-(Naphthalen-1-yl­amino)­cyclo­hexa­nol

**DOI:** 10.1107/S1600536811021714

**Published:** 2011-06-11

**Authors:** Rachid Outouch, Brahim Boualy, Mustapha Ait Ali, Larbi El Firdoussi, Corrado Rizzoli

**Affiliations:** a Equipe de Chimie de Coordination et Catalyse, Faculté des Sciences-Semlalia, BP 2390, 40001 Marrakech, Morocco; b Dipartimento di Chimica Generale ed Inorganica, Chimica Analitica, Chimica Fisica, Universitá degli Studi di Parma, Viale G. P. Usberti 17/A, I-43124 Parma, Italy

## Abstract

The title compound, C_16_H_19_NO, was synthesized under solvent-free conditions by reaction of 7-oxa-bicyclo­[4.1.0]heptane and naphthalen-1-amine in the presence of Ca(CF_3_COO)_2_ as catalyst. The cyclo­hexane ring adopts a chair conformation. In the crystal, mol­ecules are linked by inter­molecular N—H⋯O hydrogen bonds and C—H⋯π inter­actions into chains parallel to the *c* axis.

## Related literature

For background to applications of β-amino­alcohols in organic synthesis, see: Rogers *et al.* (1989 ▶); O’Brien (1999 ▶); Ager *et al.* (1996 ▶). For the synthesis of β-amino­alcohols, see: Deyrup & Moyer (1969 ▶); Kamal, Ramu *et al.* (2005 ▶); Yarapathy *et al.* (2006 ▶); Yadav *et al.* (2003 ▶); Rafiee *et al.* (2004 ▶); Robin *et al.* (2007 ▶); Das *et al.* (2000 ▶); Kamal, Adil & Arifuddin (2005 ▶). For puckering parameters, see: Cremer & Pople (1975 ▶).
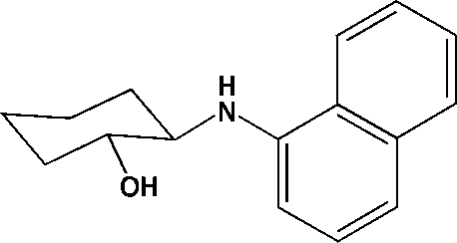


## Experimental

### 

#### Crystal data

C_16_H_19_NO
*M* *_r_* = 241.32
Orthorhombic, 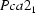

*a* = 12.0278 (4) Å
*b* = 11.5910 (3) Å
*c* = 9.5566 (3) Å
*V* = 1332.33 (7) Å^3^
*Z* = 4
Cu *K*α radiation
μ = 0.58 mm^−1^
*T* = 294 K
0.18 × 0.15 × 0.10 mm

#### Data collection

Siemens AED diffractometer
4933 measured reflections
1353 independent reflections
1326 reflections with *I* > 2σ(*I*)
*R*_int_ = 0.034
3 standard reflections every 100 reflections  intensity decay: 0.0%

#### Refinement

*R*[*F* ^2^ > 2σ(*F* ^2^)] = 0.030
*wR*(*F* ^2^) = 0.087
*S* = 1.08
1353 reflections
169 parameters
1 restraint
H atoms treated by a mixture of independent and constrained refinement
Δρ_max_ = 0.14 e Å^−3^
Δρ_min_ = −0.13 e Å^−3^

### 

Data collection: *AED* (Belletti *et al.*, 1993 ▶); cell refinement: *AED*; data reduction: *AED*; program(s) used to solve structure: *SIR97* (Altomare *et al.*, 1999 ▶); program(s) used to refine structure: *SHELXL97* (Sheldrick, 2008 ▶); molecular graphics: *ORTEP-3 for Windows* (Farrugia, 1997 ▶) and *SCHAKAL97* (Keller, 1997 ▶); software used to prepare material for publication: *SHELXL97* and *PARST95* (Nardelli, 1995 ▶).

## Supplementary Material

Crystal structure: contains datablock(s) global, I. DOI: 10.1107/S1600536811021714/zl2377sup1.cif

Structure factors: contains datablock(s) I. DOI: 10.1107/S1600536811021714/zl2377Isup2.hkl

Supplementary material file. DOI: 10.1107/S1600536811021714/zl2377Isup3.cml

Additional supplementary materials:  crystallographic information; 3D view; checkCIF report

## Figures and Tables

**Table 1:** Hydrogen-bond geometry (Å, °) *Cg*1 is the centroid of the C7–C11/C16 ring.

*D*—H⋯*A*	*D*—H	H⋯*A*	*D*⋯*A*	*D*—H⋯*A*
N1—H1*N*⋯O1^i^	0.83 (3)	2.30 (3)	3.125 (2)	171 (2)
C14—H14⋯*Cg*1^i^	0.93	2.71	3.530 (3)	148

Symmetry code: (i) 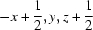.

**Fig. 1.**
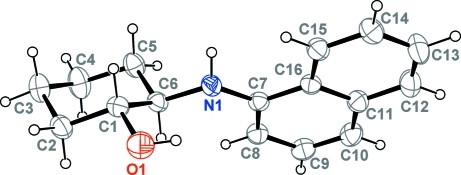
The molecular structure of the title compound, with displacement ellipsoids drawn at the 30% probability level.

**Fig. 2.**
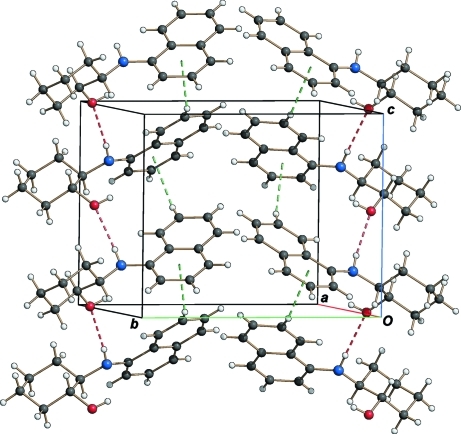
Packing diagram of the title compound showing the formation of molecular chains along the a axis via intermolecular N—H···O hydrogen bonds (red dashed lines) and C—H···π interactions (green dashed lines).

## supplementary crystallographic information

### Comment

β-Amino alcohols are useful organic intermediates owing to their versatility as building blocks in the synthesis of several biologically active natural products (Rogers *et al.*, 1989), unnatural amino acids (O'Brien, 1999) and chiral auxiliaries (Ager *et al.*, 1996). These compounds are traditionally synthesized by direct treatment of epoxides with excessive amounts of amines at elevated temperatures (Deyrup & Moyer, 1969). Under such conditions, less reactive epoxides and sluggish amines react slowly and sensitive functional groups undergo undesirable side reactions. Many alterations were made in recent years to enhance the synthetic scope of this reaction by the use of Lewis acid catalysis (Kamal, Ramu *et al.*, 2005), solid phase synthesis (Yarapathy *et al.*, 2006), ionic liquids (Yadav *et al.*, 2003), heteropolyacids (Rafiee *et al.*, 2004), microwave irradiation (Robin *et al.*, 2007), fluorinated solvents (Das *et al.*, 2000), and ultrasound mediation (Kamal, Adil & Arifuddin, 2005). As a contribution to this widespread area, we describe here the synthesis and crystal structure of the title amino alcohol.

In the molecule of the title compound (Fig. 1), the cyclohexane ring adopts a chair conformation with puckering parameters (Cremer & Pople, 1975) *Q* = 0.5765 (19) Å, θ = 2.6 (2)° and φ = 31 (5)°. The hydroxy and amine substituent to the ring are equatorially oriented. In the crystal structure (Fig. 2), intermolecular N—H···O hydrogen bonds and C—H···π interactions (Table 1) link the molecules into chains running parallel to the *c* axis.

### Experimental

In a screw capped vial equipped with a magnetic stirrer, Ca(CF_3_CO_2_)_2_ (0.03 g, 0.11 mmol) was added to naphthalen-1-amine (0.293 g, 2.04 mmol) and 7-oxa-bicyclo[4.1.0]heptane (0.481 g, 2.00 mmol), and the resulting mixture was left under vigorous stirring at 313 K (40°C) for 31 h. The mixture was extracted with AcOEt (3 × 10 ml), and the combined organic layers were dried over anhydrous Na_2_SO_4_. The combined filtrates were concentrated under vacuum to afford the title product (276 mg, yield 56%). Crystals suitable for X-ray analysis were obtained by slow evaporation of a diethyl ether solution. M.p. 366–367 K.

### Refinement

The amine H atom was located in a difference Fourier map and refined freely. All other H atoms were placed at calculated positions and refined using a riding model approximation, with C—H = 0.93–0.98 Å, O—H = 0.82 Å, and with *U*_iso_(H) = 1.2 *U*_eq_(C) or 1.5 *U*_eq_(C, O) for methyl and hydroxy H atoms. In the absence of significant anomalous scattering effects, 460 Friedel pairs were merged in the last cycles of refinement.

### Crystal data

**Table:**

C_16_H_19_NO	*F*(000) = 520
*M**_r_* = 241.32	*D*_x_ = 1.203 Mg m^−^^3^
Orthorhombic, *P**c**a*2_1_	Cu *K*α radiation, λ = 1.54178 Å
Hall symbol: P 2c -2ac	Cell parameters from 48 reflections
*a* = 12.0278 (4) Å	θ = 16.7–36.3°
*b* = 11.5910 (3) Å	µ = 0.58 mm^−^^1^
*c* = 9.5566 (3) Å	*T* = 294 K
*V* = 1332.33 (7) Å^3^	Block, pale-blue
*Z* = 4	0.18 × 0.15 × 0.10 mm

### Data collection

**Table:**

Siemens AED diffractometer	*R*_int_ = 0.034
Radiation source: fine-focus sealed tube	θ_max_ = 69.9°, θ_min_ = 3.8°
graphite	*h* = −14→13
θ/2θ scans	*k* = −14→13
4933 measured reflections	*l* = −11→5
1353 independent reflections	3 standard reflections every 100 reflections
1326 reflections with *I* > 2σ(*I*)	intensity decay: 0.0%

### Refinement

**Table:**

Refinement on *F*^2^	Secondary atom site location: difference Fourier map
Least-squares matrix: full	Hydrogen site location: inferred from neighbouring sites
*R*[*F*^2^ > 2σ(*F*^2^)] = 0.030	H atoms treated by a mixture of independent and constrained refinement
*wR*(*F*^2^) = 0.087	*w* = 1/[σ^2^(*F*_o_^2^) + (0.0541*P*)^2^ + 0.0897*P*] where *P* = (*F*_o_^2^ + 2*F*_c_^2^)/3
*S* = 1.08	(Δ/σ)_max_ < 0.001
1353 reflections	Δρ_max_ = 0.14 e Å^−^^3^
169 parameters	Δρ_min_ = −0.13 e Å^−^^3^
1 restraint	Extinction correction: *SHELXL*
Primary atom site location: structure-invariant direct methods	Extinction coefficient: 0.0102 (11)

### Special details

**Table:**

Refinement. Refinement of *F*^2^ against ALL reflections. The weighted *R*-factor *wR* and goodness of fit *S* are based on *F*^2^, conventional *R*-factors *R* are based on *F*, with *F* set to zero for negative *F*^2^. The threshold expression of *F*^2^ > σ(*F*^2^) is used only for calculating *R*-factors(gt) *etc*. and is not relevant to the choice of reflections for refinement. *R*-factors based on *F*^2^ are statistically about twice as large as those based on *F*, and *R*- factors based on ALL data will be even larger.

### Fractional atomic coordinates and isotropic or equivalent isotropic displacement parameters (Å^2^)

**Table:**

	*x*	*y*	*z*	*U*_iso_*/*U*_eq_	
O1	0.27577 (13)	−0.01165 (12)	0.00639 (19)	0.0690 (4)	
H1O	0.2788	0.0522	0.0431	0.104*	
N1	0.14586 (12)	0.08201 (12)	0.21600 (17)	0.0491 (3)	
H1N	0.1730 (16)	0.0622 (17)	0.292 (3)	0.054 (5)*	
C1	0.20467 (14)	−0.08488 (14)	0.08667 (19)	0.0471 (4)	
H1	0.2464	−0.1137	0.1675	0.057*	
C2	0.16883 (16)	−0.18640 (16)	−0.0013 (2)	0.0572 (4)	
H2A	0.2339	−0.2284	−0.0331	0.069*	
H2B	0.1288	−0.1591	−0.0829	0.069*	
C3	0.09482 (17)	−0.26593 (17)	0.0831 (3)	0.0661 (6)	
H3A	0.1366	−0.2976	0.1609	0.079*	
H3B	0.0706	−0.3296	0.0246	0.079*	
C4	−0.00636 (18)	−0.20139 (19)	0.1385 (3)	0.0747 (7)	
H4A	−0.0500	−0.2527	0.1969	0.090*	
H4B	−0.0525	−0.1774	0.0605	0.090*	
C5	0.02757 (15)	−0.09561 (16)	0.2233 (2)	0.0577 (5)	
H5A	−0.0384	−0.0529	0.2503	0.069*	
H5B	0.0651	−0.1202	0.3081	0.069*	
C6	0.10412 (13)	−0.01752 (13)	0.13964 (18)	0.0458 (4)	
H6	0.0628	0.0107	0.0582	0.055*	
C7	0.08882 (12)	0.18703 (14)	0.22162 (18)	0.0431 (3)	
C8	−0.01028 (14)	0.20562 (15)	0.1532 (2)	0.0500 (4)	
H8	−0.0435	0.1456	0.1040	0.060*	
C9	−0.06185 (15)	0.31447 (17)	0.1569 (2)	0.0583 (5)	
H9	−0.1284	0.3253	0.1091	0.070*	
C10	−0.01684 (16)	0.40383 (17)	0.2286 (3)	0.0609 (5)	
H10	−0.0527	0.4749	0.2303	0.073*	
C11	0.08457 (15)	0.38906 (15)	0.3007 (2)	0.0530 (4)	
C12	0.1337 (2)	0.47978 (17)	0.3771 (3)	0.0687 (6)	
H12	0.0984	0.5511	0.3805	0.082*	
C13	0.2316 (2)	0.46537 (17)	0.4458 (3)	0.0744 (6)	
H13	0.2618	0.5261	0.4968	0.089*	
C14	0.28733 (17)	0.35946 (17)	0.4400 (3)	0.0630 (5)	
H14	0.3550	0.3504	0.4859	0.076*	
C15	0.24238 (16)	0.26941 (14)	0.3671 (2)	0.0508 (4)	
H15	0.2803	0.1995	0.3636	0.061*	
C16	0.13946 (13)	0.28016 (13)	0.29679 (17)	0.0443 (4)	

### Atomic displacement parameters (Å^2^)

**Table:**

	*U*^11^	*U*^22^	*U*^33^	*U*^12^	*U*^13^	*U*^23^
O1	0.0746 (9)	0.0685 (8)	0.0640 (9)	−0.0070 (7)	0.0230 (8)	0.0016 (8)
N1	0.0558 (8)	0.0455 (7)	0.0460 (8)	0.0049 (6)	−0.0107 (7)	−0.0054 (7)
C1	0.0484 (8)	0.0510 (8)	0.0419 (8)	0.0012 (7)	0.0005 (7)	0.0008 (7)
C2	0.0614 (10)	0.0588 (9)	0.0513 (10)	0.0102 (8)	−0.0018 (8)	−0.0113 (9)
C3	0.0658 (11)	0.0534 (10)	0.0793 (14)	−0.0043 (8)	0.0012 (11)	−0.0181 (10)
C4	0.0556 (10)	0.0691 (13)	0.0995 (19)	−0.0144 (9)	0.0103 (12)	−0.0260 (13)
C5	0.0532 (9)	0.0604 (10)	0.0595 (11)	−0.0051 (8)	0.0087 (9)	−0.0113 (9)
C6	0.0478 (7)	0.0490 (8)	0.0407 (9)	0.0029 (6)	−0.0057 (7)	−0.0039 (7)
C7	0.0445 (7)	0.0460 (8)	0.0389 (8)	0.0010 (6)	0.0033 (7)	0.0017 (7)
C8	0.0462 (8)	0.0537 (9)	0.0501 (10)	−0.0019 (7)	−0.0029 (7)	0.0006 (8)
C9	0.0486 (9)	0.0647 (10)	0.0616 (11)	0.0069 (8)	−0.0053 (9)	0.0068 (10)
C10	0.0596 (10)	0.0545 (10)	0.0684 (12)	0.0133 (8)	0.0016 (10)	0.0020 (9)
C11	0.0580 (9)	0.0501 (8)	0.0508 (10)	0.0029 (7)	0.0059 (8)	−0.0026 (8)
C12	0.0814 (13)	0.0501 (9)	0.0747 (15)	0.0058 (9)	−0.0010 (12)	−0.0125 (10)
C13	0.0828 (15)	0.0594 (10)	0.0811 (15)	−0.0082 (10)	−0.0099 (13)	−0.0226 (12)
C14	0.0622 (10)	0.0653 (10)	0.0616 (11)	−0.0076 (9)	−0.0105 (9)	−0.0066 (10)
C15	0.0516 (8)	0.0516 (8)	0.0491 (9)	−0.0001 (8)	−0.0023 (8)	−0.0023 (8)
C16	0.0478 (8)	0.0466 (8)	0.0385 (8)	−0.0007 (6)	0.0044 (7)	−0.0003 (7)

### Geometric parameters (Å, °)

**Table:**

O1—C1	1.428 (2)	C6—H6	0.9800
O1—H1O	0.8200	C7—C8	1.377 (2)
N1—C7	1.398 (2)	C7—C16	1.433 (2)
N1—C6	1.454 (2)	C8—C9	1.406 (2)
N1—H1N	0.83 (3)	C8—H8	0.9300
C1—C2	1.509 (2)	C9—C10	1.355 (3)
C1—C6	1.526 (2)	C9—H9	0.9300
C1—H1	0.9800	C10—C11	1.411 (3)
C2—C3	1.514 (3)	C10—H10	0.9300
C2—H2A	0.9700	C11—C12	1.410 (3)
C2—H2B	0.9700	C11—C16	1.425 (2)
C3—C4	1.523 (3)	C12—C13	1.359 (4)
C3—H3A	0.9700	C12—H12	0.9300
C3—H3B	0.9700	C13—C14	1.399 (3)
C4—C5	1.526 (3)	C13—H13	0.9300
C4—H4A	0.9700	C14—C15	1.367 (3)
C4—H4B	0.9700	C14—H14	0.9300
C5—C6	1.519 (3)	C15—C16	1.414 (3)
C5—H5A	0.9700	C15—H15	0.9300
C5—H5B	0.9700		
			
C1—O1—H1O	109.5	N1—C6—C1	107.38 (13)
C7—N1—C6	122.71 (14)	C5—C6—C1	110.50 (13)
C7—N1—H1N	113.6 (15)	N1—C6—H6	108.0
C6—N1—H1N	110.9 (14)	C5—C6—H6	108.0
O1—C1—C2	109.60 (16)	C1—C6—H6	108.0
O1—C1—C6	110.40 (13)	C8—C7—N1	122.87 (15)
C2—C1—C6	110.94 (14)	C8—C7—C16	119.24 (15)
O1—C1—H1	108.6	N1—C7—C16	117.81 (14)
C2—C1—H1	108.6	C7—C8—C9	120.67 (17)
C6—C1—H1	108.6	C7—C8—H8	119.7
C1—C2—C3	110.28 (17)	C9—C8—H8	119.7
C1—C2—H2A	109.6	C10—C9—C8	121.51 (17)
C3—C2—H2A	109.6	C10—C9—H9	119.2
C1—C2—H2B	109.6	C8—C9—H9	119.2
C3—C2—H2B	109.6	C9—C10—C11	119.97 (17)
H2A—C2—H2B	108.1	C9—C10—H10	120.0
C2—C3—C4	110.83 (17)	C11—C10—H10	120.0
C2—C3—H3A	109.5	C12—C11—C10	121.65 (17)
C4—C3—H3A	109.5	C12—C11—C16	118.67 (18)
C2—C3—H3B	109.5	C10—C11—C16	119.68 (16)
C4—C3—H3B	109.5	C13—C12—C11	121.47 (19)
H3A—C3—H3B	108.1	C13—C12—H12	119.3
C3—C4—C5	111.45 (16)	C11—C12—H12	119.3
C3—C4—H4A	109.3	C12—C13—C14	120.24 (19)
C5—C4—H4A	109.3	C12—C13—H13	119.9
C3—C4—H4B	109.3	C14—C13—H13	119.9
C5—C4—H4B	109.3	C15—C14—C13	120.04 (19)
H4A—C4—H4B	108.0	C15—C14—H14	120.0
C6—C5—C4	111.16 (18)	C13—C14—H14	120.0
C6—C5—H5A	109.4	C14—C15—C16	121.42 (17)
C4—C5—H5A	109.4	C14—C15—H15	119.3
C6—C5—H5B	109.4	C16—C15—H15	119.3
C4—C5—H5B	109.4	C15—C16—C11	118.12 (15)
H5A—C5—H5B	108.0	C15—C16—C7	122.96 (14)
N1—C6—C5	114.69 (15)	C11—C16—C7	118.92 (15)

### Hydrogen-bond geometry (Å, °)

**Table:**

Cg1 is the centroid of the C7–C11/C16 ring.

**Table:**

*D*—H···*A*	*D*—H	H···*A*	*D*···*A*	*D*—H···*A*
N1—H1N···O1^i^	0.83 (3)	2.30 (3)	3.125 (2)	171 (2)
C14—H14···Cg1^i^	0.93	2.71	3.530 (3)	148

Symmetry codes: (i) −*x*+1/2, *y*, *z*+1/2.
